# Decoding Parkinson's disease: a systematic review of qEEG biomarkers across clinical domains

**DOI:** 10.1016/j.cnp.2026.10.007

**Published:** 2026-10-19

**Authors:** Marjan Makhsous, Maryam Izadpanahi, Ehsan Rezayat

**Affiliations:** a Department of Cognitive Sciences, Faculty of Psychology and Education, University of Tehran, Tehran, Iran; b Department of Neuroscience, Faculty of Medicine, Mashhad University of Medical Sciences, Mashhad, Iran

## Abstract

**Objective:**

This systematic review synthesized evidence on quantitative EEG (qEEG) correlates of Parkinson's disease (PD) across five domains: cognition, motor function, pharmacological interventions, neuromodulation, and sleep/non-motor features, evaluating qEEG's potential as a biomarker across these clinical domains.

**Methods:**

Following PRISMA, we searched PubMed, Embase, Web of Science, and Scopus for studies published from 1991 to 2025. We included studies with ≥10 idiopathic PD patients, resting-state qEEG, and empirical data. Methodological quality was assessed using JBI checklists and AMSTAR.

**Results:**

We included 89 studies. Spectral slowing – reduced posterior dominant frequency and increased theta power – was the most consistent correlate of cognitive impairment. Connectivity measures (PLV, graph metrics) showed stronger associations with motor dysfunction, gait impairment, and freezing than spectral power alone. qEEG changed with dopaminergic drugs (increased alpha/beta) and neuromodulation (DBS, tDCS, rTMS, neurofeedback). Sleep studies revealed spindle density reductions linked to dementia risk.

**Conclusions:**

qEEG is a promising biomarker for PD, with distinct electrophysiological correlates across cognitive, motor, pharmacological, and neuromodulatory domains. However, methodological standardization is needed before clinical use.

**Significance:**

With standardized protocols and multicenter validation, qEEG could aid diagnosis, progression tracking, and personalized therapy in PD.

## Highlights

• Quantitative EEG (qEEG) slowing marks cognitive decline in Parkinson's disease (PD).
• Connectivity measures better track motor dysfunction in PD.
• qEEG responds to dopaminergic therapy and neuromodulation.

## 1 Introduction

Parkinson's disease (PD) is a progressive neurodegenerative disorder that affects not only motor functions—such as tremor, rigidity, bradykinesia, and postural instability—but also a broad range of non-motor features, including cognitive decline, sleep disturbances, psychiatric comorbidities, and autonomic dysfunction (Helkala et al., 1991; Tanaka et al., 2000). These non-motor symptoms frequently emerge years before the classic motor signs and are major contributors to long-term disability and diminished quality of life.

Against this backdrop, electroencephalography (EEG) has drawn growing interest as a non-invasive means of capturing cortical activity and identifying early neural changes. Among its various applications, quantitative EEG (qEEG) stands out as a particularly attractive candidate for biomarker development in PD. A considerable body of work has now documented that patients with PD often exhibit slowing of background rhythms, alterations in spectral power, and breakdowns in functional connectivity—changes that appear most pronounced in those with cognitive involvement (Soikkeli et al., 1991; Caviness et al., 2007; Morita et al., 2009). The utility of qEEG, however, extends well beyond cognition. Beyond cognitive applications, the same technique has been used for assessing motor symptom severity, following changes induced by medication, evaluating the outcomes of neuromodulation protocols, and examining sleep-related phenomena (Guner et al., 2017; Hassan et al., 2017). Over the past several years, graph-theoretical frameworks have gradually gained traction as a means of estimating global and local network properties from EEG data, which in turn may offer clues about disease progression and help shorten the often lengthy diagnostic pathway (Utianski et al., 2016; Ricciardi et al., 2021).

At the same time, it must be acknowledged that the existing evidence is marked by substantial inconsistency. The field is characterized by a wide range of experimental designs, small and often heterogeneous samples, divergent recording parameters, and a multitude of signal processing pipelines. These factors collectively undermine the comparability of results and limit the strength of conclusions that can be drawn. In the earlier phases of research, investigations were generally carried out on relatively small patient groups recruited from individual medical centers. By contrast, the current wave of studies has shown a clear shift toward the application of machine learning techniques and more elaborate network-based analytical approaches (Fonseca et al., 2019; Novak et al., 2022). Nevertheless, despite these methodological advancements, the field has thus far lacked a comprehensive, well-structured overview that cuts across all relevant clinical domains—one that also takes a critical look at how qEEG might actually be used in practice, whether for early case detection, monitoring disease evolution over time, or informing treatment decisions.

Motivated by this recognized gap, we carried out a systematic review of qEEG studies in idiopathic PD, covering publications from 1991 up to and including 2025. The review process followed the PRISMA guidelines. To categorize and synthesize the available evidence, we drew on the domain structure originally suggested by Geraedts et al. (2018) and applied it as an organizing framework across five main clinical areas: cognition, motor function, pharmacological interventions, neuromodulation, and sleep/non-motor outcomes. The overarching question we set out to answer was straightforward: Which qEEG metrics—whether derived from spectral analysis, connectivity measures, or more advanced computational approaches—show consistent associations with cognitive decline, motor impairment, treatment responsiveness, neuromodulation effects, and non-motor symptoms in idiopathic PD? Ultimately, we aimed not only to offer a current, integrated overview of the existing evidence but also to critically weigh methodological strengths and weaknesses, and to chart a realistic path forward for both research and clinical implementation.

Note that there are a number of previous reviews that have summarized qEEG findings related to cognition in PD (Geraedts et al., 2018; Spoa et al., 2025; Zawiślak-Fornagiel et al., 2025). However, those reviews tended to concentrate on isolated clinical domains rather than adopting a broader, multi-domain perspective. To achieve a more holistic view, we made a conscious decision to preserve and revisit older studies from the 1991–2017 period alongside more recent publications. Two considerations guided this choice. On the one hand, it gave us the opportunity to examine how qEEG correlates have shifted in tandem with methodological refinements—from straightforward spectral power measurements to connectivity-based and network-oriented approaches. On the other hand, several classic observations, such as slowing of the dominant frequency and beta-band abnormalities, continue to function as benchmark reference points against which newer biomarkers are routinely assessed. By weaving together both historical and contemporary evidence within a single five-domain framework, we believe we have captured the full developmental arc of qEEG research in PD more completely than any previous domain-restricted review.

## 2 Methods

### 2.1 Search strategy

A systematic literature search was conducted to identify studies investigating quantitative EEG (qEEG) in idiopathic Parkinson's disease (PD). Following the approach of Geraedts et al. (2018), we searched PubMed, Embase, Web of Science, and Scopus for publications from January 1991 to March 2025. The search combined PD-related terms (“Parkinson's disease, “ “idiopathic PD”) with electrophysiological markers (“EEG,” “qEEG,” “spectral power,” “coherence,” “connectivity,” “dominant frequency,” “machine learning,” “wavelet,” “TBPC”) using Boolean operators. Manual screening of reference lists from relevant articles and systematic reviews was also performed (Geraedts et al., 2018).

**Full search string for PubMed**

The complete search string for PubMed (via PubMed Advanced Search Builder) was:

("Parkinson disease"[MeSH] OR "Parkinson's disease"[tiab] OR "Parkinson disease"[tiab] OR "idiopathic Parkinson"[tiab])

AND

(electroencephalography[MeSH] OR EEG[tiab] OR "quantitative EEG"[tiab] OR qEEG[tiab] OR "spectral power"[tiab] OR coherence[tiab] OR connectivity[tiab] OR "dominant frequency"[tiab] OR "machine learning"[tiab] OR wavelet[tiab] OR TBPC[tiab])

AND

("1991/01/01"[PDAT] : "2025/03/31"[PDAT])

This search was limited to human studies (AND “humans”[MeSH Terms]) and English language articles (AND English[la]). No additional filters for study design were applied to maximize sensitivity. The same strategy was adapted for Embase (using Emtree terms and /exp. syntax), Web of Science (using TS = field tags), and Scopus (using TITLE-ABS-KEY field tags). The full search strings for all databases are available in the Supplementary Materials (Table S7).

### 2.2 Eligibility criteria

Studies were included if they: (1) enrolled ≥10 adults with idiopathic PD; (2) used case-control, case series, randomized controlled trial (RCT), longitudinal cohort, or systematic review/meta-analysis designs; (3) reported resting-state EEG/qEEG data; and (4) examined associations with at least one of five domains: cognition, motor function, pharmacological interventions, neuromodulation, or sleep/non-motor outcomes. Studies were excluded if they focused on non-idiopathic parkinsonism, included fewer than 10 PD participants, were non-empirical reports, or were limited to evoked potentials without resting-state EEG (Soikkeli et al., 1991; Caviness et al., 2007). A summary of the inclusion and exclusion criteria is provided in Table 1.

**Table 1:** Inclusion and exclusion criteria.

Category	Criteria
Population	Adults diagnosed with *idiopathic Parkinson's disease*
Sample size	Minimum of **10 participants** in the PD group
Study designs (included)	Case–control studies; case series; randomized controlled trials; longitudinal cohort studies; systematic reviews/meta-analyses
EEG requirements	Resting-state EEG with quantitative analysis (spectral, connectivity, or advanced qEEG methods)
Clinical domains	Cognition; motor function; pharmacological interventions; neuromodulation; sleep and other non-motor outcomes
Exclusion: population	Atypical or secondary parkinsonism (e.g., MSA, PSP, vascular parkinsonism)
Exclusion: sample size	Fewer than 10 PD participants
Exclusion: EEG type	Studies using only evoked potentials (ERP) or task-based EEG without resting-state data
Exclusion: study type	Non-empirical reports (editorials, letters, commentaries)
Exclusion: data quality	Studies lacking extractable qEEG data or insufficient methodological detail

### 2.3 Study selection

All identified records were imported into reference management software for deduplication. Two independent reviewers screened titles and abstracts, followed by full-text assessment. Disagreements were resolved by consensus. After full-text assessment, 89 studies met the inclusion criteria. The detailed breakdown of study designs and time periods is provided in the Results section and Fig. 1 (PRISMA flow diagram). Reference lists of relevant articles and systematic reviews were also screened manually to supplement the database search.

**Fig. 1:**
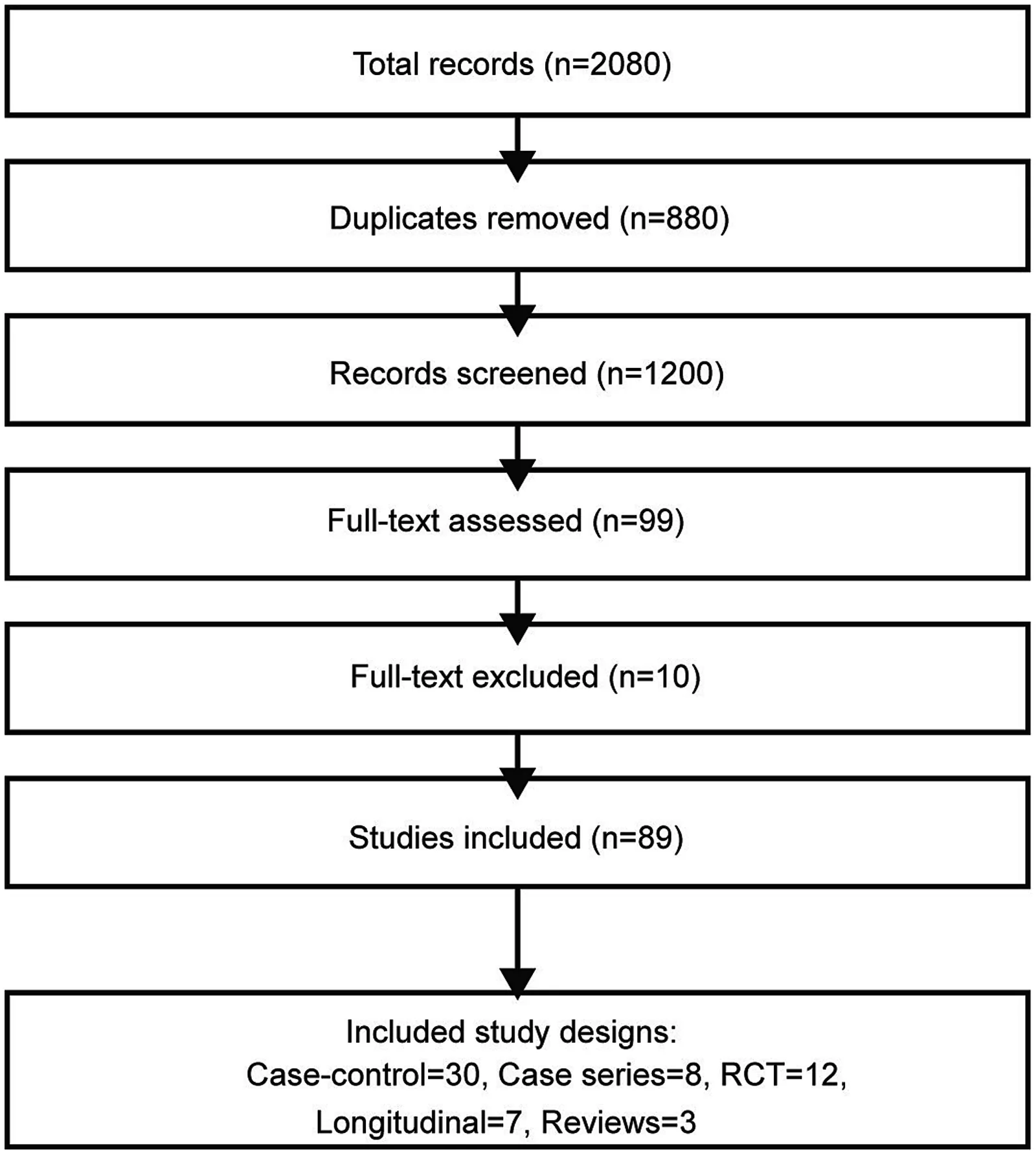
PRISMA 2020 flow diagram for study selection. A total of 2080 records were identified across PubMed, Embase, Web of Science, and Scopus. After removing 880 duplicates, 1200 records were screened by title and abstract. Of these, 99 full-text articles were assessed for eligibility, and 10 were excluded for the following reasons: non-idiopathic parkinsonism (*n* = 3), no resting-state EEG data (*n* = 2), sample size <10 participants (*n* = 2), no quantitative EEG outcomes (*n* = 2), and non-empirical report (*n* = 1). A total of 89 studies were included in the final review. Study designs included case-control (*n* = 30), observational/cross-sectional (*n* = 23), randomized controlled trials (*n* = 12), case series (*n* = 8), longitudinal cohorts (*n* = 7), experimental (*n* = 4), systematic reviews (*n* = 3), and cross-over (*n* = 2). The full search strategy is provided in the Methods section.

### 2.4 Data extraction

A structured data extraction form was used to collect bibliographic details, study characteristics, clinical profile, qEEG methodology (spectral power, dominant frequency, coherence, connectivity, advanced analytics), and domain classification according to the five predefined clinical areas (see Results and Tables S1–S5 for detailed distribution). Two reviewers independently extracted data, resolving discrepancies by consensus.

### 2.5 Quality assessment

Methodological quality was evaluated using Joanna Briggs Institute (JBI) checklists appropriate to each study design and AMSTAR for systematic reviews. Scores ranged from 6 to 10 depending on the checklist used, with no study rated as high risk of bias (see Supplementary Table S6 for full details). Methodological rigor was assessed independently by two reviewers, with disagreements settled through discussion.

### 2.6 Data synthesis

A qualitative synthesis approach was employed. Extracted evidence was organized within the five predefined domains. Findings were integrated narratively according to qEEG measure type (spectral, connectivity-based, advanced analytics), highlighting consistent patterns, methodological heterogeneity, and emerging biomarkers (Vecchio et al., 2013; Ricciardi et al., 2021). The distribution of included studies across the five clinical domains examined in this review is summarized in Fig. 2.

**Fig. 2:**
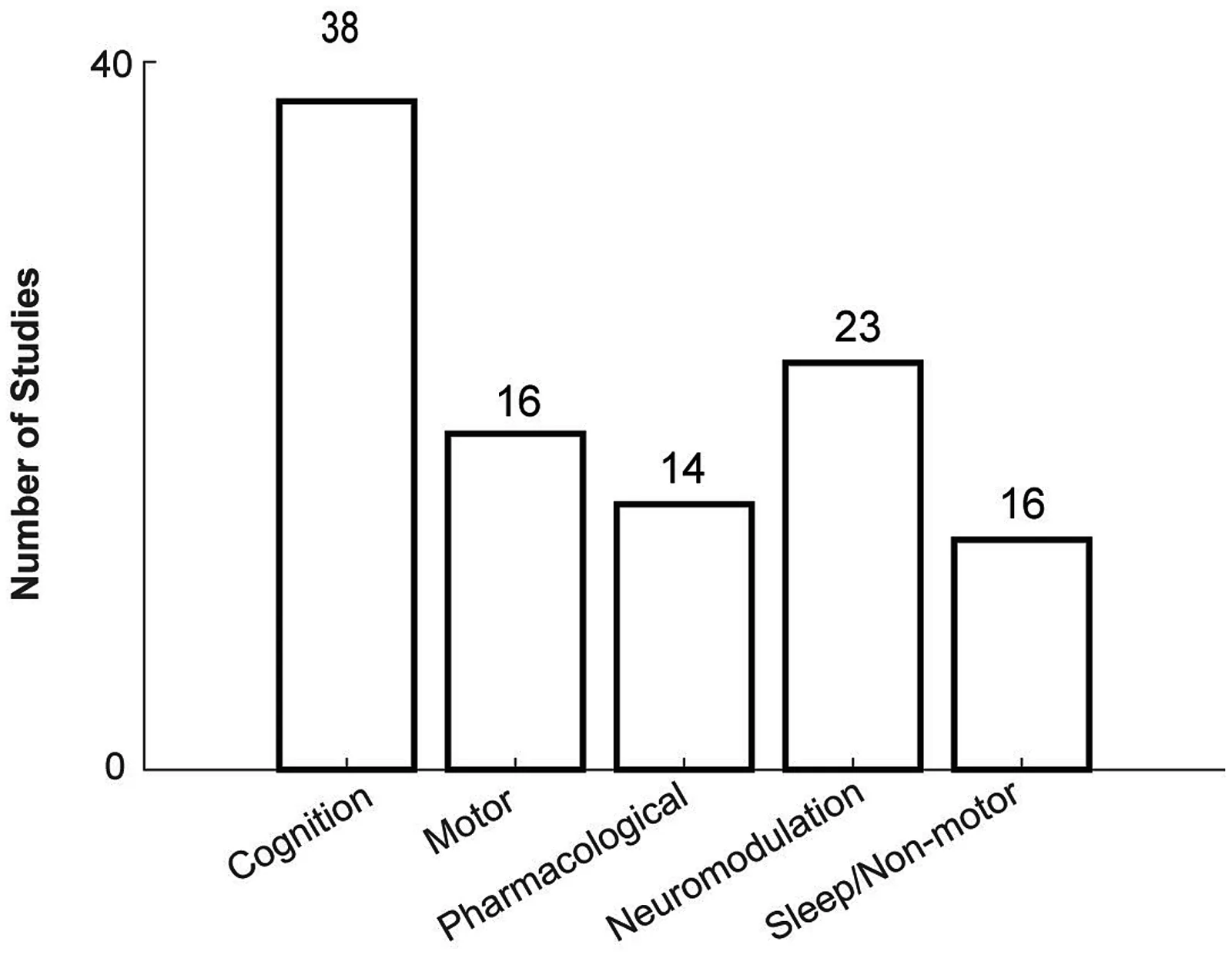
Distribution of included studies across clinical domains. Bar chart showing the number of included studies addressing each of the five clinical domains examined in this review: cognition (*n* = 38), motor function (*n* = 16), pharmacological interventions (*n* = 14), neuromodulation (*n* = 23), and sleep/non-motor features (*n* = 16). Note that some studies reported findings relevant to more than one domain and are therefore counted in more than one category.

## 3 Results

### 3.1 Search results and study characteristics

A total of 89 studies met the inclusion criteria and were included in the final review (see Methods for full search strategy and selection process). Of these, 50 were published between 1991 and 2017 and 39 were published between 2018 and 2025. The studies included a range of designs including case-control, case series, RCTs, longitudinal cohorts and systematic reviews with sample sizes ranging from 12 to over 130 patients and disease stages from early to severe PD. The five predefined clinical domains and the corresponding number of studies in each are summarized in Tables S1–S5.A broad range of qEEG metrics was reported across the included studies. These included conventional spectral measures such as band power and dominant frequency, as well as coherence and asymmetry indices. Connectivity was assessed using phase-based and graph-theoretical approaches, including PLI, PLV, EWCI, and MST. Several investigations also applied more advanced analytical strategies, among them machine learning classifiers, wavelet-based transformations, and time-burst connectivity analysis (TBPC).

To evaluate methodological quality, we used JBI checklists specific to each study design, along with AMSTAR for the systematic reviews. Across the 89 included studies, the risk of bias was predominantly low or moderate, and no study was categorized as high risk (Supplementary Table S6). Analytical, cross-sectional, and network-based studies consistently met the highest proportion of criteria (typically scoring 8 out of 8). Studies from before 2010, particularly those with case-control or case-series designs, tended to score in the moderate-risk category — typically between 6 and 7 out of 10 — with points lost mainly due to incomplete adjustment for key confounders such as disease duration or medication status. When it came to RCTs testing neuromodulation, scores generally fell between 9 and 10 out of 13. The main reasons for losing points were insufficient blinding and unclear allocation concealment, rather than any fundamental flaw in study design. We also assessed two systematic reviews using the AMSTAR checklist — Spoa et al. (2025) and Zawiślak-Fornagiel et al. (2025) — both of which met most of the criteria, though each had minor shortcomings in how publication bias was handled. The PRISMA flow diagram (Fig. 1) provides a visual summary of how the final set of studies was selected.

### 3.2 Cognition

Cognitive function in PD was explored in 38 studies. Among these, twelve identified a correlation between increased EEG slowing (increased delta/theta power) and more severe cognitive impairment, defined by decreased global cognitive scores or deficits in specific domains (Soikkeli et al., 1991; Caviness et al., 2007; Caviness et al., 2011; Morita et al., 2009; Helkala et al., 1991; Tanaka et al., 2000; Pozzi et al., 1994; Neufeld et al., 1994; Guner et al., 2017; Kamei et al., 2010; Eichelberger et al., 2017). Patients with dementia demonstrated marked increases in delta/theta power and reductions in alpha/beta power compared to cognitively normal PD patients (Soikkeli et al., 1991; Caviness et al., 2007; Morita et al., 2009).

Five studies reported a positive correlation between fast–slow spectral ratio and cognitive performance, although one study found that the association depended on the specific cognitive test used (Morita et al., 2009; Caviness et al., 2011; Klassen et al., 2011; Tanaka et al., 2000; Pozzi et al., 1994). Four of five studies found that a higher dominant frequency correlated with better cognition, although one reported mixed results (Klassen et al., 2011; Caviness et al., 2011; Tanaka et al., 2000; Pozzi et al., 1994). EEG background slowing similarly distinguished PD patients with mild cognitive impairment or dementia from cognitively normal PD patients across independent cohorts (Benz et al., 2014; Bousleiman et al., 2014; Chaturvedi et al., 2017; Zimmermann et al., 2015). Reduced resting-state EEG complexity and lower alpha/theta ratios at baseline were associated with poorer neuropsychological performance and predicted subsequent cognitive decline (Keller et al., 2020; Jaramillo-Jimenez et al., 2021; Arnaldi et al., 2017). Machine-learning classifiers trained on EEG features achieved good discrimination of cognitive impairment severity (Betrouni et al., 2019), while reduced cholinergic basal forebrain volume was linked to diminished posterior alpha reactivity in patients with mild cognitive impairment (Rea et al., 2021).

Findings for beta power were inconsistent: three studies reported a positive correlation with cognition (Tanaka et al., 2000; Caviness et al., 2011; Helkala et al., 1991), while six found no significant relationship (Soikkeli et al., 1991; Caviness et al., 2007; Morita et al., 2009; Neufeld et al., 1994; Guner et al., 2017; Eichelberger et al., 2017).

Connectivity-based analyses provided further insights. Graph-theoretical metrics revealed reduced synchronization and network integration, particularly in the alpha band, among cognitively impaired PD patients (Utianski et al., 2016). Mostile et al. (2019) also investigated alterations in resting-state EEG network activity in patients with Parkinson's disease and mild cognitive impairment (Mostile et al., 2019). A larger study using PLV and EWCI found lower alpha1/alpha2 edge-wise connectivity in cognitively impaired patients, with EWCI—but not global PLV—correlating with cognitive scores (Hassan et al., 2017). Simpler measures such as signal asymmetry did not correlate with global cognition (Utianski et al., 2016).

Recent studies confirmed these observations. Zawiślak-Fornagiel et al. (2023) reported increased theta power and a reduced alpha/theta ratio in Parkinson's disease patients with dementia (Zawiślak-Fornagiel et al., 2023). Posterior rhythm slowing below 8.5 Hz predicted dementia progression (Vecchio et al., 2013; Vecchio et al., 2016; Vecchio et al., 2017; Vecchio et al., 2021). Machine learning approaches achieved high accuracy in classifying cognitive subgroups (Fonseca et al., 2019; Fonseca et al., 2020; Fonseca et al., 2022). Oscillatory slowing differentiated motor phenotypes and predicted cognitive risk (Ricciardi et al., 2020; Ricciardi et al., 2021). Dynamic connectivity metrics such as TBPC predicted cognitive decline (Gschwandtner et al., 2023), and wavelet-based qEEG indices discriminated cognitive subgroups (Novak et al., 2022). Two systematic reviews confirmed posterior rhythm slowing and elevated theta power as the most frequent qEEG markers of cognitive impairment (Spoa et al., 2025; Zawiślak-Fornagiel et al., 2025). The main associations between qEEG metrics and cognitive status across the included studies are summarized in Fig. 3.

**Fig. 3:**
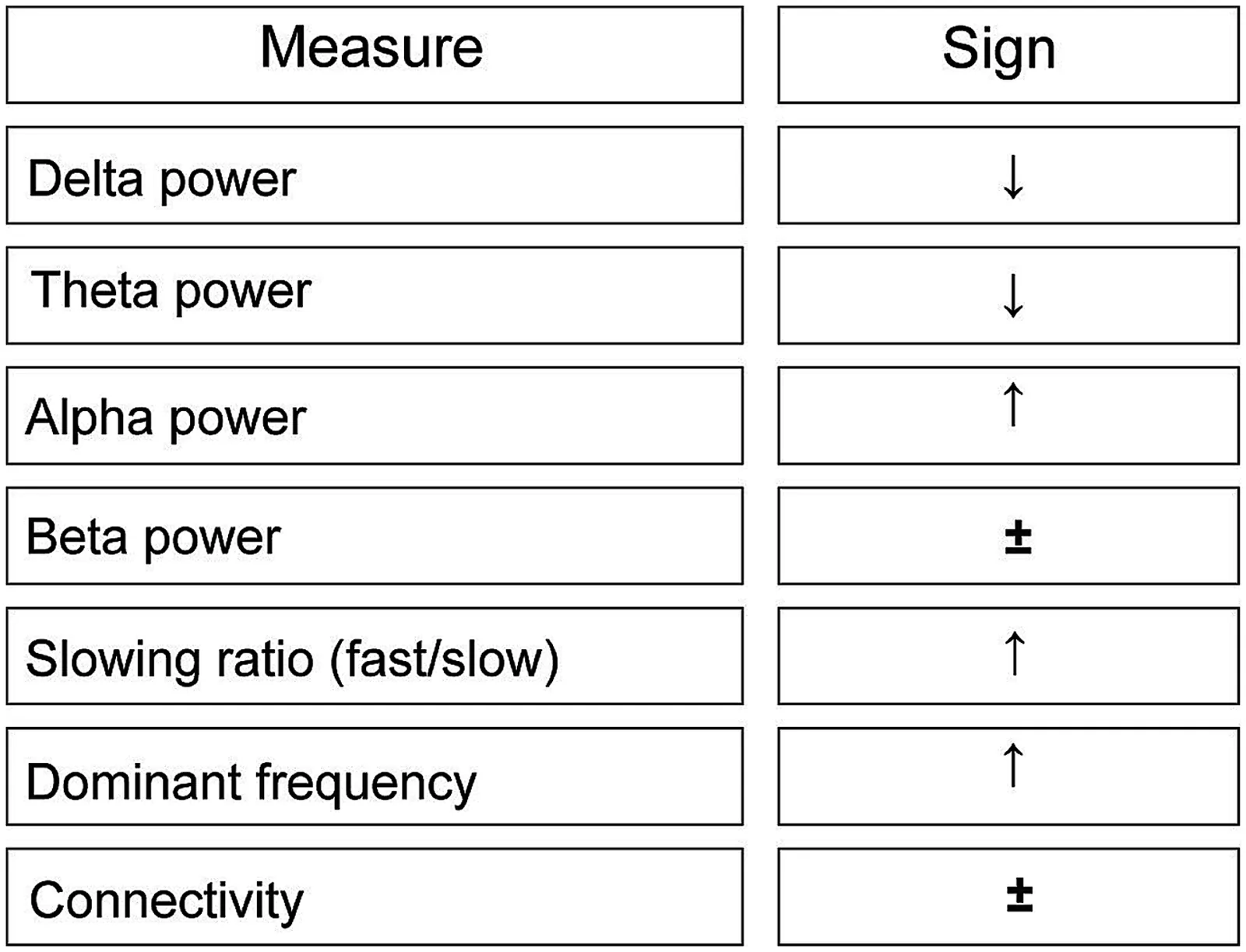
Overview of correlations between qEEG spectral and connectivity measurements and cognitive status in Parkinson's disease. Increased in delta and theta power and decreased in alpha power were consistently associated with cognitive impairment. Beta power has shown mixed results in different studies. Higher fast/slow ratios and dominant frequency were positively correlated with cognition ability. Connectivity measures demonstrated variable associations depending on the measure used.

### 3.3 Longitudinal cognitive assessment

Five longitudinal studies examined qEEG as a predictor of cognitive trajectory. Caviness et al. (2015) reported an association between longitudinal changes in EEG delta-band power and deterioration in cognitive performance in Parkinson's disease (Caviness et al., 2015). Lower baseline dominant frequency predicted cognitive decline in three cohorts, while higher baseline theta power predicted conversion to dementia (Klassen et al., 2011; Caviness et al., 2016; Morita et al., 2011). MEG studies documented progressive decline in network topology over three years (Olde Dubbelink et al., 2014). TBPC profiles predicted cognitive decline with AUC ≈ 0.80 (Gschwandtner et al., 2023). Wavelet-based indices (increased RTF-theta, decreased RTF-beta) discriminated cognitive subgroups with AUC ≈ 0.89 (Novak et al., 2022). The predictive value of baseline qEEG measurements for longitudinal cognitive decline is summarized in Fig. 5.

### 3.4 Motor function

Sixteen studies explored associations between qEEG and motor severity. Four studies found no significant correlation between spectral power and MDS-UPDRS III or Hoehn and Yahr stage (Soikkeli et al., 1991; Caviness et al., 2007; Caviness et al., 2016; Helkala et al., 1991). Acute levodopa increased alpha/beta power and improved UPDRS scores (Melgari et al., 2014), while long-term therapy produced heterogeneous spectral changes (Stanzione et al., 1996; Morita et al., 2009).

Dominant frequency negatively correlated with rigidity (Jech et al., 2006). Fast–slow spectral ratio correlated negatively with Hoehn and Yahr stage (Morita et al., 2009; Morita et al., 2011). Specific associations included correlations between HY stage and alpha-2 amplitude, beta power, and theta power (Caviness et al., 2012; He et al., 2017; Caviness et al., 2016). Beta coherence correlated with UPDRS III in one study but not replicated in early-onset PD (George et al., 2013; He et al., 2017). EEG asymmetry findings were mixed (Mostile et al., 2015).

Longitudinal studies did not support qEEG as a predictor of motor progression (Klassen et al., 2011; Caviness et al., 2016).

Connectivity-based measures showed stronger associations: freezing of gait (Bosch et al., 2022), motor asymmetry (Rajagopal et al., 2025), and gait impairment (Di Biase et al., 2023). Network-level analyses revealed small-world disruption (Vecchio et al., 2021). Eye-state sensitivity and qEEG risk indices predicted motor risk (Giménez-Aparisi et al., 2023; Liu et al., 2022). The associations between qEEG measurements and motor function across the included studies are shown in Fig. 6.

### 3.5 Pharmacological interventions

Fourteen studies assessed the sensitivity of qEEG to dopaminergic treatment. Two studies reported no long-lasting spectral changes following oral treatment with levodopa or dopamine agonists (Stanzione et al., 1996; Morita et al., 2009). In contrast, one study observed increases in alpha and beta power within sixty minutes after levodopa administration, which correlated with improvements in MDS-UPDRS III scores (Melgari et al., 2014). Another investigation noted a reduction in beta coherence after dopamine therapy (George et al., 2013). Two studies reported that baseline spectral slowing does not predict long-term motor progression, indicating limited prognostic value for these pharmacological qEEG markers (Caviness et al., 2016; Klassen et al., 2011).

Recent studies have extended these findings. Increases in delta power during dopamine treatment have been shown to predict cognitive decline, suggesting effects beyond the motor system (Saleh et al., 2021). Levodopa has been reported to alter alpha and beta rhythms associated with verbal fluency (Hatz et al., 2019), and restoration of EEG microstates following dopaminergic modulation has been observed (Lamoš et al., 2023). Additionally, dopamine treatment has been shown to increase sleep spindle activity, suggesting a link between pharmacology and sleep architecture (Memon et al., 2023a, Memon et al., 2023b, Memon et al., 2023c). Eye-state sensitivity has been highlighted as a complementary biomarker responsive to dopaminergic modulation (Giménez-Aparisi et al., 2023), and qEEG during therapy can predict motor risk (Liu et al., 2022). Furthermore, oscillatory slowing has been shown to differentiate motor phenotypes under dopaminergic influence (Ricciardi et al., 2020). The responsiveness of qEEG measures to pharmacological and neuromodulatory interventions is illustrated in Fig. 4.

**Fig. 4:**
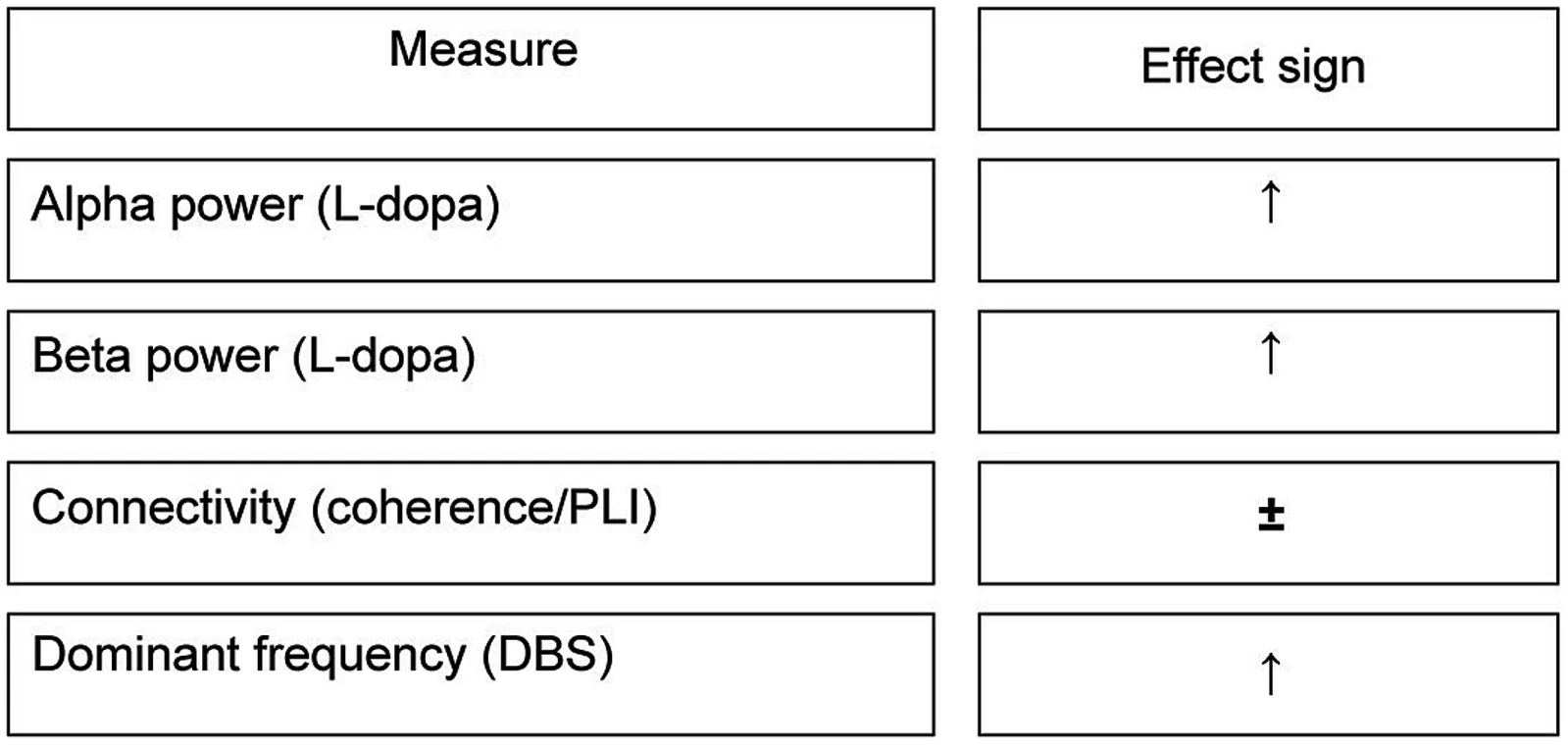
Responsiveness of qEEG measurements to interventions in Parkinson's disease. Short-term dopaminergic treatment increased alpha and beta power, and deep brain stimulation increased dominant frequency and beta activity. Connectivity measures (e.g., coherence, PLI) demonstrated variable changes. Long-term oral dopaminergic therapy has shown heterogeneous spectrum of effects without consistent patterns.

### 3.6 Neuromodulation

Twenty-three studies investigated the effects of neuromodulation techniques—including DBS, tDCS, tACS, tRNS, rTMS, and neurofeedback—on qEEG measurements in PD.

**DBS:** Eight studies examined DBS effects on cortical rhythms. Classic reports described modulation of dominant frequency and beta power. Jech (2006) noted an increase in dominant frequency when stimulation was “on,” (Jech et al., 2006) and Swann et al. (2011) recorded increased frontal and parietal beta power during task performance. Caviness et al. (2016) found that spectral slowing did not predict motor progression. More recent research expanded this picture: increased delta power predicted cognitive decline in DBS cohorts (Saleh et al., 2021); alterations in alpha/beta rhythms were associated with verbal fluency (Hatz et al., 2019); DBS increased sleep spindle activity (Memon et al., 2023a, Memon et al., 2023b, Memon et al., 2023c); and restoration of EEG microstates was observed (Lamoš et al., 2023).

**tDCS, tACS, tRNS:** Ten studies investigated non-invasive electrical stimulation. Five RCTs reported improvements in motor outcomes or working memory following tDCS, although two had mixed results (Benninger et al., 2010; Boggio et al., 2006; Fregni et al., 2006; Lefaucheur et al., 2004; Kober et al., 2019). Recent EEG-guided protocols demonstrated targeted modulation and clinical improvements (Cantarella et al., 2019; Del Felice et al., 2021). EEG-guided tRNS/tACS improved motor performance (Hosseini et al., 2023; Zoghi et al., 2022), and cerebellar gamma tACS enhanced gait and cognition (Grover et al., 2023).

**rTMS:** Eleven studies, including one meta-analysis, examined rTMS protocols. Early investigations found that high-frequency rTMS over M1 facilitated motor function (Cantello et al., 1991; Ridding et al., 1995; Pascual-Leone et al., 1994; Shimamoto et al., 2001; Khedr et al., 2003). Three studies targeting DLPFC improved depression and executive function (Elahi et al., 2009; Wu et al., 2010; Pal et al., 2010). Later work showed that baseline oscillatory features predicted motor outcomes (Lee et al., 2019; Yang et al., 2018). A meta-analysis summarized protocol-dependent variability (Shirota et al., 2013).

**Neurofeedback:** Four experimental studies demonstrated that EEG-guided neurofeedback targeting beta-band modulation improved motor smoothness, reduced rigidity, and enhanced movement initiation (Marzbani et al., 2017; Gharabaghi et al., 2014; Naseri et al., 2022; Khatami et al., 2022).

### 3.7 Sleep and other non-motor domains

Sixteen studies investigated qEEG correlates of sleep and non-motor features.

Sleep: Nine studies examined sleep architecture. PD patients with RBD exhibited increased delta/theta power and reduced spindle density (Brunner et al., 2002; Iranzo et al., 2004; Caviness et al., 2007; Kotagal et al., 2012). NREM spectral slowing predicted dementia conversion (Massicotte-Marquez et al., 2008; De Cock et al., 2011). Decreased spindle density correlated with cognitive impairment (Kotagal et al., 2012). DBS increased spindle activity (Memon et al., 2023a, Memon et al., 2023b, Memon et al., 2023c). Sleep qEEG predicted motor risk (Liu et al., 2022). Network disruption during sleep was observed (Vecchio et al., 2021). Greater slow-wave sleep and higher NREM delta power were associated with better cognitive performance (Wood et al., 2021), while lower baseline sleep spindle density predicted the later development of dementia (Latreille et al., 2015).

**Other Non-Motor Domains:** Dopamine therapy altered alpha/beta rhythms associated with verbal fluency (Hatz et al., 2019). Increased delta power during treatment predicted cognitive decline (Saleh et al., 2021). Oscillatory slowing differentiated motor phenotypes and correlated with psychiatric symptoms (Ricciardi et al., 2020). Eye-state sensitivity emerged as a novel biomarker (Giménez-Aparisi et al., 2023).

### 3.8 Summary of findings

Across 89 studies, qEEG demonstrated consistent domain-specific associations with cognition, motor function, pharmacological responses, neuromodulation effects, and sleep/non-motor features in Parkinson's disease. Spectral slowing, dominant frequency alterations, and connectivity metrics were the most frequently reported correlates, with modern analytical approaches refining these patterns. Detailed study characteristics, methodological quality ratings, and domain-specific quantitative summaries are presented in Tables S1–S6, and corresponding visual syntheses are shown in Fig. 1, Fig. 2, Fig. 3, Fig. 4, Fig. 5, Fig. 6.

**Fig. 5:**
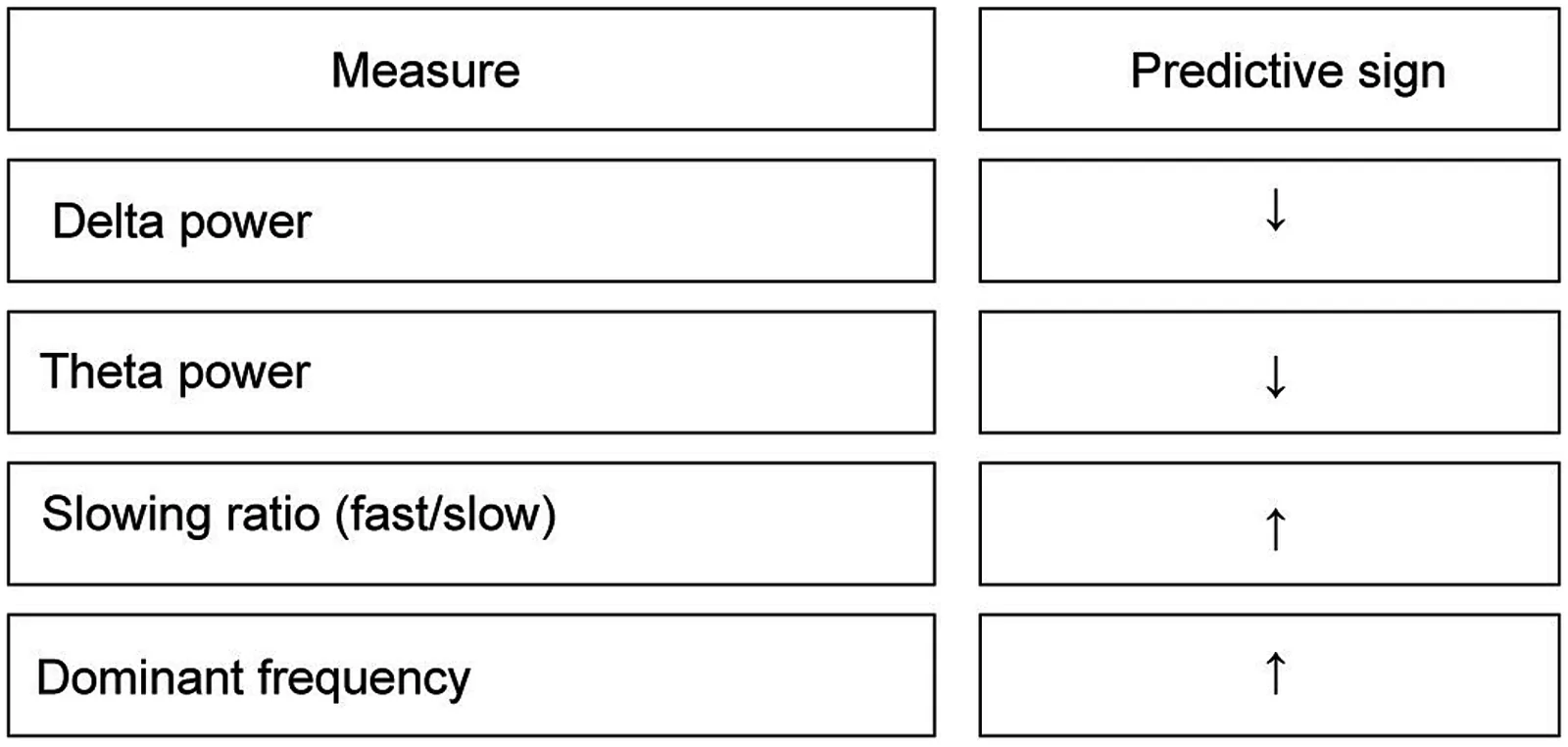
Predictive value of baseline qEEG measurements for longitudinal cognitive decline in Parkinson's disease. Increased delta and theta power predicted poorer cognition, whereas higher fast/slow and dominant frequency ratios were protective. These findings highlight that qEEG decline is as a biomarker of future cognitive decline.

**Fig. 6:**
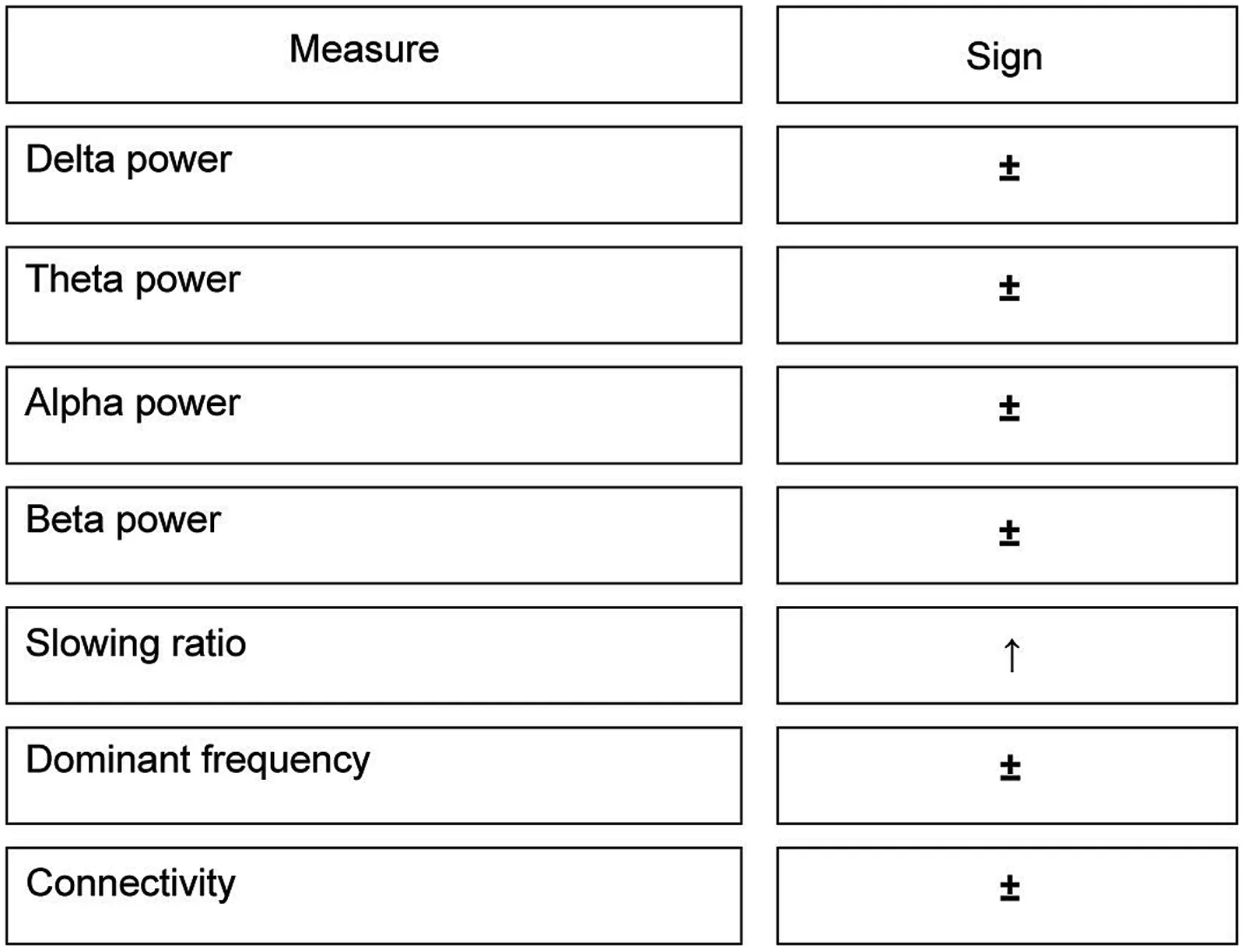
Summary of the correlation between qEEG measurements and motor function in Parkinson's disease. Associations were inconsistent across studies. Some reports indicated positive correlations of fast/slow ratios and alpha/beta power with motor improvement, while others found no significant association. Key measures of frequency and connectivity showed mixed results, with network-level measures showing stronger relationships.

## 4 Discussion

Quantitative EEG (qEEG) provides a non-invasive window into cortical dysfunction in Parkinson's disease (PD), and findings across three decades of research converge on several robust electrophysiological signatures. Among these, spectral slowing—manifested as reduced posterior dominant frequency and increased theta power—emerges as the most consistent correlate of cognitive impairment. Early cross-sectional studies repeatedly demonstrated strong associations between slowing and global cognitive deficits (Soikkeli et al., 1991; Caviness et al., 2007; Morita et al., 2009), while longitudinal cohorts confirmed that lower baseline dominant frequency and elevated theta power predict future cognitive decline and conversion to dementia (Klassen et al., 2011; Caviness et al., 2016; Morita et al., 2011). Additional evidence linking increased theta power to anosmia and subsequent cognitive deterioration (Cozac et al., 2016a, Cozac et al., 2016b; Cozac et al., 2017) further supports the prognostic value of spectral slowing.

Recent methodological advances have expanded the scope of qEEG beyond classical spectral metrics. Machine-learning classifiers have achieved high accuracy in distinguishing cognitive subgroups (Fonseca et al., 2019; Fonseca et al., 2020; Fonseca et al., 2022), while connectivity-based analyses have revealed network-level disruptions—particularly within alpha-band networks—that differentiate clinical phenotypes and predict cognitive risk (Ricciardi et al., 2020; Ricciardi et al., 2021). Dynamic connectivity measures such as TBPC (Gschwandtner et al., 2023) and wavelet-based indices (Novak et al., 2022) have demonstrated strong discriminative performance (AUC ≈ 0.80–0.90), highlighting the potential of advanced analytics to refine early detection of cognitive decline. Altered connectivity has also been associated with apathy and other neuropsychiatric symptoms (Hatz et al., 2017), suggesting that qEEG captures broader aspects of cortical network dysfunction.

In contrast to cognitive findings, motor-related qEEG results remain more heterogeneous. Classical studies reported inconsistent associations between spectral power and motor severity (Soikkeli et al., 1991; Caviness et al., 2007; Caviness et al., 2016), and longitudinal cohorts did not support spectral slowing as a predictor of motor progression (Klassen et al., 2011; Caviness et al., 2016). Short-term levodopa administration reliably increased alpha and beta power in parallel with motor improvement (Melgari et al., 2014), but these effects were not sustained with long-term therapy (Stanzione et al., 1996; Morita et al., 2009). Findings related to coherence and asymmetry were similarly inconsistent, with some studies reporting associations with rigidity or disease stage (George et al., 2013; Mostile et al., 2015) and others finding no significant relationships (He et al., 2017). These discrepancies suggest that spectral power alone is insufficient to characterize motor dysfunction.

Consequently, recent research has shifted toward connectivity-based qEEG metrics, which show stronger and more specific associations with motor features. Measures such as phase-locking value, network synchrony, and graph-theoretical indices have been linked to rigidity, gait impairment, motor asymmetry, and freezing of gait (Bosch et al., 2022; Rajagopal et al., 2025; Di Biase et al., 2023). Network-level analyses have identified small-world deficits in PD (Vecchio et al., 2021), suggesting reduced cortical efficiency as a substrate of motor impairment. Emerging approaches—including eye-state sensitivity (Giménez-Aparisi et al., 2023) and qEEG-based motor risk indices (Liu et al., 2022)—further highlight the potential of network-level biomarkers for motor profiling.

Neuromodulation studies reinforce qEEG's sensitivity to therapeutic modulation. Deep brain stimulation (DBS) alters dominant frequency and beta activity (Jech et al., 2006; Swann et al., 2011), while more recent work shows DBS-related changes in slow rhythms, spindle activity, and microstate dynamics (Saleh et al., 2021; Memon et al., 2023a, Memon et al., 2023b, Memon et al., 2023c; Lamoš et al., 2023). Non-invasive stimulation techniques—including tDCS, tACS, and tRNS—have demonstrated improvements in motor and cognitive outcomes, particularly when stimulation parameters are individualized using EEG-guided protocols (Cantarella et al., 2019; Del Felice et al., 2021; Hosseini et al., 2023). rTMS studies similarly report protocol-dependent effects, with baseline oscillatory features predicting treatment response (Lee et al., 2019; Yang et al., 2018). Neurofeedback interventions consistently show improvements in motor performance through self-modulation of beta-band activity (Marzbani et al., 2017; Naseri et al., 2022).

Sleep-related qEEG abnormalities provide additional insight into non-motor dysfunction. Patients with REM sleep behavior disorder (RBD) exhibit increased delta/theta power and reduced spindle density (Brunner et al., 2002; Iranzo et al., 2004; Kotagal et al., 2012), and NREM spectral slowing predicts conversion to dementia (Massicotte-Marquez et al., 2008; De Cock et al., 2011). DBS increases spindle activity (Memon et al., 2023a, Memon et al., 2023b, Memon et al., 2023c), and sleep qEEG features predict motor risk (Liu et al., 2022). Network-level disruptions extend qEEG biomarkers beyond spectral power, linking oscillatory slowing and alpha/beta modulation to psychiatric and autonomic dysfunction (Ricciardi et al., 2020; Giménez-Aparisi et al., 2023).

Taken together, these findings carry direct clinical relevance. EEG slowing is not just a statistical curiosity—it appears to track with something real and clinically meaningful in the brain. The consistent link between reduced posterior dominant frequency, increased theta activity, and poorer cognitive performance suggests that when we see this pattern in a patient, we should be thinking about the integrity of cortical networks, particularly those supporting attention and memory. Importantly, this association is not confined to patients who already have dementia. Even in non-demented individuals, baseline slowing seems to carry prognostic weight, raising the possibility that serial qEEG recordings could one day help flag those at higher risk of future cognitive decline—well before standard neuropsychological tests show obvious changes.

When it comes to motor symptoms, the picture is more nuanced, and that in itself is instructive. The fact that spectral power measures alone have yielded inconsistent correlations with motor severity tells us that the neural signatures of motor dysfunction are probably more distributed and network-dependent than a simple shift in local oscillatory power would capture. This is where connectivity-based metrics come into their own. The stronger associations observed between measures like phase locking value, graph-theoretic indices, and specific motor features—such as freezing of gait or asymmetry—point toward a model in which motor impairment in PD arises not from a single cortical locus but from disruptions in the way different regions coordinate their activity. From a clinical standpoint, this shift in emphasis matters: it suggests that if we want to monitor motor function or predict deterioration, we may need to look beyond the traditional spectral bands and start thinking in terms of network efficiency and inter-regional communication.

Beyond the cognitive and motor findings, pharmacological and neuromodulation studies offer a different kind of insight — one that bears more directly on how qEEG might be used at the bedside. Consider the levodopa studies: several of them showed that qEEG shifts can be picked up within minutes of drug administration, and that those shifts track quite closely with the degree of motor improvement. That kind of temporal sensitivity raises an intriguing possibility — that EEG could one day function as a real-time indicator of whether a given treatment is actually working, rather than relying solely on clinical observation or patient self-report.

This is particularly attractive in settings where clinical assessment is subjective or where patients have difficulty reporting their symptoms accurately. Similarly, the fact that different neuromodulation protocols—DBS, tDCS, rTMS—produce distinguishable qEEG signatures raises the possibility of using EEG not just as an outcome measure but as a guide for parameter selection and individualization of therapy. In other words, rather than applying a one-size-fits-all stimulation protocol, clinicians might one day use a patient's baseline oscillatory profile to predict which approach is most likely to work, and then track the effects in real time.

The sleep related findings, though fewer in number, carry their own clinical weight. The association between reduced spindle density, NREM slowing, and dementia risk suggests that the sleeping brain may be particularly revealing of underlying neurodegenerative processes. This is clinically useful because sleep EEG is relatively easy to obtain and does not require active patient cooperation, making it feasible even in more disabled individuals. If these markers prove to be robust predictors in larger, prospective cohorts, they could offer a low-burden screening tool for cognitive risk in PD. None of these applications are yet ready for routine clinical use. Several factors currently limit this translation. The heterogeneity we have repeatedly highlighted—in recording montages, reference choices, frequency band definitions, and analytical pipelines—is not merely a methodological nuisance. It has direct clinical consequences because it means that results obtained in one center may not generalize to another, and that normative reference values are difficult to establish. This is a major barrier to regulatory approval and clinical adoption. Moreover, most studies to date have been cross-sectional, and even the longitudinal ones have relatively short follow-up periods. We still know little about how qEEG trajectories evolve over years rather than months, and whether early changes are truly predictive or merely coincidental.

Looking ahead, the path toward clinical translation will require a coordinated effort across multiple fronts. Standardization of acquisition and analysis protocols is the first and most obvious step. But beyond that, we need large, multicenter longitudinal studies that track patients from the premotor stage through advanced disease, with concurrent clinical, cognitive, and neuroimaging assessments. Such studies would allow us to determine not only whether qEEG predicts outcomes, but also how early the predictive signal emerges and how specific it is to PD versus other neurodegenerative conditions. The integration of machine learning and dynamic connectivity measures is promising, but these approaches need to be validated in independent datasets before they can be trusted in clinical decision-making.

In summary, the evidence reviewed here supports the view that qEEG is more than an academic exercise. It captures genuine, biologically meaningful signals that relate to cognitive status, motor function, treatment effects, and sleep architecture in PD. The correlations are not perfect, and the heterogeneity is real, but the consistency of certain findings—particularly spectral slowing for cognition and connectivity measures for motor features—suggests that we are looking at a set of tools that, with further refinement, could genuinely improve patient care. The challenge now is to move from proof-of-principle studies to pragmatic, standardized, and validated applications that can withstand the scrutiny of clinical practice.

## 5 Conclusion

Resting-state quantitative EEG provides a scalable and non-invasive window into the neurophysiological alterations underlying Parkinson's disease. Across more than three decades of research, spectral slowing—particularly reduced posterior dominant frequency and increased theta power—emerged as the most reliable electrophysiological correlate of cognitive impairment (Soikkeli et al., 1991; Caviness et al., 2007; Morita et al., 2009). These qEEG features also demonstrated predictive value for future cognitive decline in longitudinal cohorts (Klassen et al., 2011; Caviness et al., 2016), supporting their potential role as early markers of neurodegeneration.

Motor-related qEEG findings were more heterogeneous, with inconsistent associations between spectral power and clinical severity. In contrast, connectivity-based and network-level measures showed stronger sensitivity for characterizing motor and non-motor dysfunction, including gait impairment, asymmetry, and freezing of gait (Bosch et al., 2022; Rajagopal et al., 2025; Di Biase et al., 2023). These findings highlight the importance of moving beyond simple spectral metrics toward more integrative models of cortical network organization.

qEEG also demonstrated responsiveness to pharmacological and neuromodulatory interventions. Short-term levodopa increased alpha/beta power in parallel with motor improvement (Melgari et al., 2014), while EEG-guided stimulation protocols—including tDCS, tACS, and DBS—modulated oscillatory dynamics and showed promise for individualized therapy (Cantarella et al., 2019; Memon et al., 2023a, Memon et al., 2023b, Memon et al., 2023c). These treatment-sensitive signatures underscore qEEG's potential utility for monitoring therapeutic response and guiding personalized interventions.

Despite these strengths, methodological variability—including differences in acquisition protocols, electrode density, reference schemes, and analytic pipelines—remains a major barrier to clinical translation. Future research should prioritize standardized acquisition, multicenter validation, and integration of advanced analytic approaches such as machine learning, dynamic connectivity, and graph-theoretical modeling (Fonseca et al., 2022; Ricciardi et al., 2021). With continued refinement and rigorous validation, qEEG has the potential to evolve into an actionable biomarker for diagnosis, prognosis, and precision medicine in Parkinson's disease.

## CRediT authorship contribution statement

**Marjan Makhsous:** Conceptualization, Data curation, Formal analysis, Investigation, Methodology, Writing – original draft, Writing – review & editing. **Maryam Izadpanahi:** Data curation, Validation, Visualization, Writing – review & editing. **Ehsan Rezayat:** Conceptualization, Supervision, Project administration, Methodology, Writing – review & editing.

## Ethical approval

As this study is a systematic review of previously published literature, no ethical approval or informed consent was required.

## Funding

This research received no specific grant from any funding agency in the public, commercial, or not-for-profit sectors.

## Declaration of competing interest

The authors declare that they have no known competing financial interests or personal relationships that could have appeared to influence the work reported in this paper.

## Appendix A Supplementary data

Supplementary material Supplementary Table S1-S7: study characteristics, quality assessments, and data base search strategies for the systematic review of qEEG biomarkers in parkinson disease.
