# Supplementary material for: Decoding Parkinson's disease: a systematic review of qEEG biomarkers across clinical domains
Source: Clin Neurophysiol Pract. 2026 Oct 19;11:900–8. doi: 10.1016/j.cnp.2026.10.007 (PMC13721006; doi:10.1016/j.cnp.2026.10.007)
Supplement: Supplementary file 1 [file mmc1.docx]

**Supplementary Material**

**Table S1 – Cognition Detailed characteristics of 38 studies investigating qEEG correlates of cognitive function in Parkinson’s disease.**

| **Author (Year)** | **Country** | **Design** | **Sample size** | **PD stage** | **Intervention / Focus** | **qEEG role** | **Domain** |
| --- | --- | --- | --- | --- | --- | --- | --- |
| Soikkeli (1991) | Finland | Case-control | 20 PD, 20 HC | Early | Resting EEG | Spectral slowing | Cognition |
| Helkala (1991) | Finland | Case-control | 18 PD | Mixed | Slow-wave activity | Cognitive dysfunction | Cognition |
| Pozzi (1994) | Argentina | Case-control | 47 PD | Mixed | Dementia vs non-dementia | Slowing | Cognition |
| Neufeld (1994) | Israel | Case series | 20 PD | Mixed | Frequency analysis | Demented vs non | Cognition |
| Tanaka (2000) | Switzerland | Case-control | 29 PD | Mixed | ERP + EEG | Dementia vs non-dementia | Cognition |
| Morita (2009, 2011) | Japan | Case series | 100+ PD | Mixed | EEG slowing | Progression & cognition | Cognition |
| Caviness (2007, 2011, 2012, 2016) | USA | Case-control / Longitudinal | 66–134 PD | Mixed | EEG slowing | Dementia progression | Cognition |
| Klassen (2011) | USA | Longitudinal | 106 PD | Early–Moderate | Theta/Alpha ratio | Dementia prediction | Cognition |
| Guner (2017) | Turkey | Case-control | 45 PD | Mixed | Early cognitive impairment | qEEG contribution | Cognition |
| Kamei (2010) | Japan | Case series | 32 PD | Mixed | Executive dysfunction | qEEG correlation | Cognition |
| Eichelberger (2017) | Switzerland | Case series | 57 PD | Mixed | Visuospatial | Slowing correlation | Cognition |
| Fonseca (2019, 2020, 2022) | Brazil | Cross-sectional / ML | 32–45 PD | Mixed | Resting EEG + ML | Cognitive classification | Cognition |
| Ricciardi (2020, 2021) | Italy | Observational | 50–60 PD | Mixed | Oscillatory slowing / Alpha-band networks | Phenotype differentiation / Cognitive risk | Cognition |
| Vecchio (2013, 2016, 2017, 2021) | Italy | Meta-analysis / Observational | Multiple | Mixed | Posterior dominant rhythm | Prognosis | Cognition |
| Gschwandtner (2023) | Switzerland | Longitudinal | 75 PD, 72 HC | Non-demented | TBPC | Predict cognitive decline | Cognition |
| Novak (2022) | USA | Observational | 20 PD, 20 HC | Mixed | Wavelet qEEG | Cognitive subgroup discrimination | Cognition |
| Zawiślak-Fornagiel (2025) | Poland | Systematic review | Multiple | Mixed | qEEG + ML | Cognitive impairment | Cognition |
| Spoa (2025) | Italy | Systematic review | Multiple | Mixed | Resting EEG | Cognitive decline | Cognition |
| Utianski (2016) | USA | Observational | 30 PD | Mixed | Graph theory metrics | Reduced synchronization | Cognition |
| Hassan (2017) | USA | Observational | 40 PD | Mixed | PLV, EWCI | Connectivity correlates with cognition | Cognition |
| Olde Dubbelink (2014) | Netherlands | Longitudinal (MEG) | 30 PD | Mixed | Network topology | Progressive decline | Cognition |
| Lamoš (2023) | Czech | Observational | 25 PD | Mixed | EEG microstates | Restoration with therapy | Cognition |
| Dubbelink (2013) | Netherlands | Longitudinal | 40 PD | Mixed | MEG connectivity | Decline over 3 years | Cognition |
| Babiloni (2011) | Italy | Observational | 50 PD | Mixed | EEG coherence | Cognitive impairment markers | Cognition |
| Keller (2020) | Switzerland | Case-control | 42 PD / 24 HC | Non-demented PD | Baseline EEG complexity (entropy) | Predicted longitudinal cognitive decline | Cognition |
| Benz (2014) | Switzerland | Case-control | PD-MCI=15, PDD=5, PD-NC, AD, HC | Mixed (MCI/PDD) | EEG background slowing vs cognitive status | Slowing distinguished PD-MCI/PDD from PD-NC | Cognition |
| Bousleiman (2014) | Switzerland | Case-control | 53 PD | Early-stage PD | Regional relative power spectra | Power spectra screened for PD-MCI | Cognition |
| Latreille (2015) | Canada | Longitudinal | 82 PD / controls | Non-demented at baseline | Sleep spindle & slow-wave density (NREM) | Lower spindle density predicted later dementia | Cognition / Sleep |
| Zimmermann (2015) | Switzerland | Cross-sectional | 48 PD | Non-demented PD | EEG slowing vs cognitive domain scores | Slowing correlated with attention, executive, fluency, memory | Cognition |
| Chaturvedi (2017) | Switzerland | Case-control | 50 PD / 41 HC | PD with/without MCI | High-resolution QEEG feature selection | QEEG measures distinguished PD from HC | Cognition |
| Betrouni (2019) | France | Observational / ML | 118 PD | Varying cognitive severity | EEG + data-mining classification models | ML models classified cognitive impairment severity | Cognition |
| Jaramillo-Jimenez (2021) | Colombia | Case-control | PD / HC (Medellin cohort) | Non-demented PD | Resting-state alpha/theta ratio | Alpha/theta ratio related to neuropsych performance | Cognition |
| Cozac (2016b) | Switzerland | Longitudinal | 37 PD | Non-demented at baseline | GRMP theta power, 3-year follow-up | Baseline theta power predicted severe cognitive decline at 3 yrs | Cognition |
| Arnaldi (2017) | Italy | Longitudinal | 54 de novo PD | De novo, drug-naive | Resting EEG + DAT-SPECT | Posterior qEEG mean frequency predicted cognitive worsening | Cognition |
| Rea (2021) | UK / Slovenia | Case-control | 31 PD / 21 MCI / 21 HC | PD without dementia | qEEG vs cholinergic basal forebrain volume (MRI) | cBF volume correlated with alpha reactivity in PD | Cognition |
| Mostile (2019) | Italy | Case-control | PD-MCI vs PD-NC (PaCoS) | Non-demented PD | Resting EEG network activity (PaCoS study) | Reduced occipital alpha, increased frontal low-freq in PD-MCI | Cognition |
| Zawiślak-Fornagiel (2023) | Poland | Case-control | 98 PD (3 cognitive groups) | PD-CogN / PD-N / PD-D | Spectral EEG vs cognitive group | Increased theta, decreased alpha/theta ratio in PD-D | Cognition |
| Caviness (2015) | USA | Longitudinal | 71 PD | Non-demented at baseline | Longitudinal QEEG vs neuropsych change | Delta bandpower change correlated with cognitive deterioration | Cognition |

**Table S2 – Motor Function Detailed characteristics of 16 studies examining qEEG markers of motor function in Parkinson’s disease.**

| **Author (Year)** | **Country** | **Design** | **Sample size** | **PD stage** | **Intervention / Focus** | **qEEG role** | **Domain** |
| --- | --- | --- | --- | --- | --- | --- | --- |
| Caviness (2012, 2016) | USA | Case-control | 66–134 PD | Mixed | Motor severity | Spectral correlations | Motor |
| He (2017) | China | Case-control | 40 PD | Mixed | Motor severity | Beta/theta correlations | Motor |
| Mostile (2015) | Italy | Case-control | 30 PD | Mixed | Motor asymmetry | EEG asymmetry | Motor |
| Bosch (2022) | Netherlands | Observational | 30 PD | Moderate | EEG connectivity | Freezing of gait | Motor |
| Rajagopal (2025) | India | Observational | 25 PD | Moderate | Phase synchronization | Motor asymmetry | Motor |
| Di Biase (2023) | Italy | Observational | 40 PD | Moderate | High-density EEG | Altered PLV networks | Motor |
| Vecchio (2021) | Italy | Observational | 35 PD | Moderate | Graph theory | Small-world disruption | Motor |
| Liu (2022) | China | Observational | 40 PD | Moderate | qEEG indices | Motor risk | Motor |
| George (2013) | USA | Case-control | 16 PD | Mixed | Dopamine therapy | Beta coherence decrease | Motor |
| Hassan (2017) | USA | Observational | 40 PD | Mixed | Connectivity metrics | Motor correlates | Motor |
| Utianski (2016) | USA | Observational | 30 PD | Mixed | Graph theory | Motor network disruption | Motor |
| Olde Dubbelink (2014) | Netherlands | Longitudinal (MEG) | 30 PD | Mixed | Network topology | Motor decline | Motor |
| Spoa (2025) | Italy | Systematic review | Multiple | Mixed | EEG slowing | Motor dysfunction | Motor |
| Zawiślak-Fornagiel (2025) | Poland | Systematic review | Multiple | Mixed | qEEG + ML | Motor impairment | Motor |
| Novak (2022) | USA | Observational | 20 PD, 20 HC | Mixed | Wavelet qEEG | Motor subgroup discrimination | Motor |
| Gschwandtner (2023) | Switzerland | Longitudinal | 75 PD, 72 HC | Non-demented | TBPC | Motor decline prediction | Motor |

**Table S3 – Pharmacological Interventions Detailed characteristics of 14 studies assessing qEEG sensitivity to pharmacological interventions in Parkinson’s disease.**

| **Author (Year)** | **Country** | **Design** | **Sample size** | **PD stage** | **Intervention / Focus** | **qEEG role** | **Domain** |
| --- | --- | --- | --- | --- | --- | --- | --- |
| Stanzione (1996) | Italy | Case-control | 19 PD | Mixed | L-dopa | Spectral change | Pharmacological |
| Morita (2009) | Japan | Case series | 100+ PD | Mixed | Long-term therapy | Spectral variability | Pharmacological |
| Melgari (2014) | Italy | Case series | 24 PD | Mixed | Acute L-dopa | Alpha/beta increase | Pharmacological |
| George (2013) | USA | Case-control | 16 PD | Mixed | Dopamine therapy | Beta coherence decrease | Pharmacological |
| Hatz (2019) | Switzerland | Cross-over | 16 PD | Advanced | DBS on/off | Alpha/beta modulation | Pharmacological |
| Saleh (2021) | Switzerland | Case-control | 16 DBS, 15 HC | Advanced | DBS | Delta ↑ predicts decline | Pharmacological |
| Caviness (2016) | USA | Longitudinal | 134 PD | Mixed | Dopamine therapy | No long-term spectral change | Pharmacological |
| Klassen (2011) | USA | Longitudinal | 106 PD | Mixed | Dopamine therapy | Limited prognostic value | Pharmacological |
| Lamoš (2023) | Czech | Observational | 25 PD | Mixed | EEG microstates | Restoration with therapy | Pharmacological |
| Memon (2023) | USA | Cross-over | 15 PD | Advanced | DBS & sleep | ↑ spindles | Pharmacological |
| Giménez-Aparisi (2023) | Spain | Observational | 25 PD | Early | Eye state sensitivity | Biomarker | Pharmacological |
| Ricciardi (2020) | Italy | Observational | 40 PD | Mixed | Oscillatory slowing | Differentiates phenotypes | Pharmacological |
| Spoa (2025) | Italy | Systematic review | Multiple | Mixed | EEG slowing | Pharmacological effects | Pharmacological |
| Zawiślak-Fornagiel (2025) | Poland | Systematic review | Multiple | Mixed | qEEG + ML | Pharmacological markers | Pharmacological |

**Table S4 – Neuromodulation** Detailed characteristics of 23 studies evaluating qEEG changes induced by neuromodulation techniques in Parkinson’s disease.

| **Author (Year)** | **Country** | **Design** | **Sample size** | **PD stage** | **Intervention / Focus** | **qEEG role** | **Domain** |
| --- | --- | --- | --- | --- | --- | --- | --- |
| Jech (2006) | Czech | Case series | 12 PD | Mixed | STN-DBS on/off | Dominant frequency change | Neuromodulation |
| Swann (2011) | USA | Case-control | 15 PD | Mixed | STN-DBS effects | ↑ beta power during task | Neuromodulation |
| Khanna (2015–18) | USA | Observational | 40–45 PD | Mixed | DBS efficacy | qEEG biomarkers | Neuromodulation |
| Caviness (2016) | USA | Longitudinal | 134 PD | Mixed | DBS cohort | Spectral slowing not predictive | Neuromodulation |
| Saleh (2021) | Switzerland | Case-control | 16 DBS, 15 HC | Advanced | DBS | Delta ↑ predicts cognitive decline | Neuromodulation |
| Hatz (2019) | Switzerland | Cross-over | 16 PD | Advanced | DBS verbal fluency | Alpha/beta modulation | Neuromodulation |
| Memon (2023) | USA | Cross-over | 15 PD | Advanced | DBS & sleep | ↑ spindles | Neuromodulation |
| Lamoš (2023) | Czech | Observational | 25 PD | Mixed | DBS | EEG microstates restoration | Neuromodulation |
| Benninger (2010) | USA | RCT | 25 PD | Moderate | tDCS | Motor improvement | Neuromodulation |
| Boggio (2006) | Brazil | RCT | 20 PD | Moderate | tDCS | Cognitive improvement | Neuromodulation |
| Fregni (2006–07) | Brazil | RCT | 20–36 PD | Moderate | tDCS (M1) | Motor outcomes | Neuromodulation |
| Lefaucheur (2004–05) | France | RCT | 32 PD | Moderate | rTMS (M1) | Motor improvement | Neuromodulation |
| Kober (2018–19) | Austria | RCT | 20–30 PD | Moderate | DLPFC tDCS | Working memory | Neuromodulation |
| Cantarella (2019–21) | Italy | Pilot RCT | 18–30 PD | Moderate | EEG-guided tDCS | Targeted stimulation | Neuromodulation |
| Del Felice (2019–21) | Italy | Pilot RCT | 18–25 PD | Moderate | Personalized tACS | EEG-guided stimulation | Neuromodulation |
| Hosseini (2023) | Iran | Pilot RCT | 20 PD | Moderate | EEG-guided tRNS | Motor improvement | Neuromodulation |
| Zoghi (2022) | Australia | Pilot RCT | 22 PD | Moderate | EEG-guided tACS/tRNS | Clinical improvement | Neuromodulation |
| Grover (2023) | India | Experimental | 28 PD | Moderate | Gamma tACS | Gait & cognition | Neuromodulation |
| Lee (2019–20) | Korea | RCT | 34–40 PD | Moderate | rTMS | Baseline EEG predicts outcomes | Neuromodulation |
| Yang (2018–19) | China | RCT | 35–40 PD | Moderate | rTMS | Motor improvement | Neuromodulation |
| Shirota (2013) | Japan | Meta-analysis | 20 studies | Mixed | rTMS | Protocol-dependent variability | Neuromodulation |
| Marzbani (2017–18) | Iran | Experimental | 24–30 PD | Mixed | Neurofeedback (beta-band) | Motor performance | Neuromodulation |
| Naseri (2022) | Iran | RCT | 25 PD | Mixed | Neurofeedback | Motor fluency | Neuromodulation |

**Table S5 – Sleep & Non-Motor Detailed characteristics of 16 studies investigating qEEG correlates of sleep and non-motor outcomes in Parkinson’s disease.**

| **Author (Year)** | **Country** | **Design** | **Sample size** | **PD stage** | **Intervention / Focus** | **qEEG role** | **Domain** |
| --- | --- | --- | --- | --- | --- | --- | --- |
| Brunner (2002) | Switzerland | Case-control | 20 PD | Mixed | Sleep EEG | RBD spectral slowing | Sleep |
| Iranzo (2004) | Spain | Case-control | 25 PD | Mixed | Sleep EEG | RBD spindle reduction | Sleep |
| Kotagal (2012) | USA | Case-control | 30 PD | Mixed | Sleep EEG | Spindle density ↓, cognition correlation | Sleep |
| Massicotte-Marquez (2008) | Canada | Case-control | 25 PD | Mixed | NREM slowing | Predicts dementia | Sleep |
| De Cock (2011) | Belgium | Case-control | 28 PD | Mixed | NREM slowing | Dementia conversion | Sleep |
| Vecchio (2021) | Italy | Observational | 35 PD | Mixed | Sleep EEG | Small-world network disruption | Sleep |
| Memon (2023) | USA | Cross-over | 15 PD | Advanced | DBS & sleep | ↑ spindles | Sleep |
| Liu (2022) | China | Observational | 40 PD | Mixed | Sleep qEEG | Predicts motor risk | Sleep |
| Schreiner (2023) | Switzerland | Observational | 25 PD | Mixed | Sleep spindles | Linked to sleepiness | Sleep |
| Terranova (2024) | Italy | Case-control | 30 PD | Mixed | REM sleep EEG | Predicts RBD onset | Sleep |
| Filipovic (1998) | Serbia | Case series | 24 PD | Mixed | Depression | ↓ alpha power | Non-motor |
| Giménez-Aparisi (2023) | Spain | Observational | 25 PD | Early | Eye state sensitivity | Biomarker | Non-motor |
| Memon (2023a) | USA | Case-control | 56 PD / 30 HC | PD, unspecified stage | NREM slow-wave & spindle characteristics | Reduced SW density in PD vs controls | Sleep |
| Memon (2023b) | USA | Observational | PD with/without RBD | PD, unspecified stage | Sleep qEEG vs RBD status | Distinguished PD-RBD from PD-no-RBD on sleep qEEG | Sleep |
| Latreille (2015) | Canada | Longitudinal | 82 PD / controls | Non-demented at baseline | Sleep spindle & slow-wave density (NREM) | Lower spindle density predicted later dementia | Cognition / Sleep |
| Wood (2021) | USA | Observational | 32 PD | PD, unspecified stage | Polysomnography + EEG delta power in N3 | Higher SWS/delta power associated with better cognition | Sleep / Cognition |

**Table S6. Quality Assessment of Included Studies (JBI Checklists)**

| **Study** | **Design** | **Checklist** | **Items Met** | **Total Score** | **Risk of Bias** |
| --- | --- | --- | --- | --- | --- |
| Soikkeli (1991) | Case-control | JBI Case-Control | 7/10 | 7 | Moderate |
| Helkala (1991) | Case-control | JBI Case-Control | 6/10 | 6 | Moderate |
| Pozzi (1994) | Case-control | JBI Case-Control | 7/10 | 7 | Moderate |
| Neufeld (1994) | Case series | JBI Case Series | 6/10 | 6 | Moderate |
| Tanaka (2000) | Case-control | JBI Case-Control | 7/10 | 7 | Moderate |
| Morita (2009) | Case series | JBI Case Series | 7/10 | 7 | Moderate |
| Morita (2011) | Case series | JBI Case Series | 7/10 | 7 | Moderate |
| Caviness (2007) | Case-control | JBI Case-Control | 8/10 | 8 | Low |
| Caviness (2011) | Case-control | JBI Case-Control | 8/10 | 8 | Low |
| Guner (2017) | Case-control | JBI Case-Control | 7/10 | 7 | Moderate |
| Kamei (2010) | Case series | JBI Case Series | 7/10 | 7 | Moderate |
| Eichelberger (2017) | Case series | JBI Case Series | 7/10 | 7 | Moderate |
| Fonseca (2019) | Cross-sectional | JBI Analytical Cross-sectional | 8/8 | 8 | Low |
| Fonseca (2020) | Cross-sectional | JBI Analytical Cross-sectional | 8/8 | 8 | Low |
| Ricciardi (2020) | Observational | JBI Analytical Cross-sectional | 8/8 | 8 | Low |
| Ricciardi (2021) | Observational | JBI Analytical Cross-sectional | 8/8 | 8 | Low |
| Vecchio (2013) | Observational | JBI Analytical Cross-sectional | 7/8 | 7 | Low |
| Vecchio (2016) | Observational | JBI Analytical Cross-sectional | 7/8 | 7 | Low |
| Vecchio (2017) | Observational | JBI Analytical Cross-sectional | 7/8 | 7 | Low |
| Vecchio (2021) | Observational | JBI Analytical Cross-sectional | 8/8 | 8 | Low |
| Gschwandtner (2023) | Longitudinal | JBI Cohort | 8/11 | 8 | Low |
| Caviness (2012) | Case-control | JBI Case-Control | 8/10 | 8 | Low |
| He (2017) | Case-control | JBI Case-Control | 7/10 | 7 | Moderate |
| Mostile (2015) | Case-control | JBI Case-Control | 7/10 | 7 | Moderate |
| Bosch (2022) | Observational | JBI Analytical Cross-sectional | 8/8 | 8 | Low |
| Rajagopal (2025) | Observational | JBI Analytical Cross-sectional | 8/8 | 8 | Low |
| Di Biase (2023) | Observational | JBI Analytical Cross-sectional | 8/8 | 8 | Low |
| Liu (2022) | Observational | JBI Analytical Cross-sectional | 8/8 | 8 | Low |
| George (2013) | Case-control | JBI Case-Control | 7/10 | 7 | Moderate |
| Hassan (2017) | Observational | JBI Analytical Cross-sectional | 8/8 | 8 | Low |
| Utianski (2016) | Observational | JBI Analytical Cross-sectional | 7/8 | 7 | Moderate |
| Olde Dubbelink (2014) | Longitudinal MEG | JBI Cohort | 7/11 | 7 | Moderate |
| Novak (2022) | Observational | JBI Analytical Cross-sectional | 8/8 | 8 | Low |
| Stanzione (1996) | Case-control | JBI Case-Control | 6/10 | 6 | Moderate |
| Melgari (2014) | Case series | JBI Case Series | 8/10 | 8 | Low |
| Hatz (2019) | Cross-over | JBI Quasi-experimental | 7/9 | 7 | Moderate |
| Saleh (2021) | Case-control | JBI Case-Control | 8/10 | 8 | Low |
| Caviness (2016) | Longitudinal | JBI Cohort | 7/11 | 7 | Moderate |
| Lamoš (2023) | Observational | JBI Analytical Cross-sectional | 8/8 | 8 | Low |
| Memon (2023) | Cross-over | JBI Quasi-experimental | 7/9 | 7 | Moderate |
| Giménez-Aparisi (2023) | Observational | JBI Analytical Cross-sectional | 8/8 | 8 | Low |
| Spoa (2025) | Systematic review | AMSTAR | 8/11 | 8 | Moderate |
| Zawiślak-Fornagiel (2025) | Systematic review | AMSTAR | 9/11 | 9 | Low |
| Benninger (2010) | RCT | JBI RCT | 9/13 | 9 | Moderate |
| Boggio (2006) | RCT | JBI RCT | 9/13 | 9 | Moderate |
| Fregni (2006–2007) | RCT | JBI RCT | 9/13 | 9 | Moderate |
| Lefaucheur (2004–2005) | RCT | JBI RCT | 9/13 | 9 | Moderate |
| Kober (2018–2019) | RCT | JBI RCT | 10/13 | 10 | Low |
| Khanna (2015–2018) | Observational | JBI Analytical Cross-sectional | 8/8 | 8 | Low |
| Grover (2023) | Experimental | JBI Quasi-experimental | 8/9 | 8 | Low |
| Hosseini (2023) | Pilot RCT | JBI RCT | 9/13 | 9 | Moderate |
| Zoghi (2022) | Pilot RCT | JBI RCT | 9/13 | 9 | Moderate |
| Naseri (2022) | RCT | JBI RCT | 10/13 | 10 | Low |
| Khatami (2022) | Experimental | JBI Quasi-experimental | 8/9 | 8 | Low |
| Marzbani (2017–2018) | Experimental | JBI Quasi-experimental | 8/9 | 8 | Low |
| Gharabaghi (2014–2016) | Experimental | JBI Quasi-experimental | 8/9 | 8 | Low |
| Cantarella (2019–2021) | RCT / Pilot | JBI RCT | 10/13 | 10 | Low |
| Del Felice (2019–2021) | Pilot RCT | JBI RCT | 10/13 | 10 | Low |
| Lee (2019–2020) | RCT | JBI RCT | 10/13 | 10 | Low |
| Yang (2018–2019) | RCT | JBI RCT | 10/13 | 10 | Low |
| Jech (2006) | Case series | JBI Case Series | 7/10 | 7 | Moderate |
| Swann (2011) | Case-control | JBI Case-Control | 7/10 | 7 | Moderate |
| Brunner (2002) | Case-control | JBI Case-Control | 7/10 | 7 | Moderate |
| Iranzo (2004) | Case-control | JBI Case-Control | 7/10 | 7 | Moderate |
| Kotagal (2012) | Case-control | JBI Case-Control | 8/10 | 8 | Low |
| Massicotte-Marquez (2008) | Case-control | JBI Case-Control | 7/10 | 7 | Moderate |
| De Cock (2011) | Case-control | JBI Case-Control | 7/10 | 7 | Moderate |
| Schreiner (2023) | Observational | JBI Analytical Cross-sectional | 8/8 | 8 | Low |
| Terranova (2024) | Case-control | JBI Case-Control | 8/10 | 8 | Low |
| Filipovic (1998) | Case series | JBI Case Series | 6/10 | 6 | Moderate |
| Gagnon (2004) | Case-control | JBI Case-Control | 7/10 | 7 | Moderate |
| Latreille (2015) | Longitudinal | JBI Cohort | 7/11 | 7 | Moderate |
| Stefani (2020) | Review | AMSTAR | 8/11 | 8 | Moderate |
| Keller (2020) | Case-control | JBI Case-Control | 8/10 | 8 | Low |
| Benz (2014) | Case-control | JBI Case-Control | 7/10 | 7 | Moderate |
| Bousleiman (2014) | Case-control | JBI Case-Control | 8/10 | 8 | Low |
| Memon (2023a) | Case-control | JBI Case-Control | 8/10 | 8 | Low |
| Memon (2023b) | Observational | JBI Analytical | 7/8 | 7 | Moderate |
| Zimmermann (2015) | Cross-sectional | JBI Analytical | 7/8 | 7 | Moderate |
| Chaturvedi (2017) | Case-control | JBI Case-Control | 8/10 | 8 | Low |
| Betrouni (2019) | Observational / ML | JBI Analytical | 7/8 | 7 | Moderate |
| Jaramillo-Jimenez (2021) | Case-control | JBI Case-Control | 8/10 | 8 | Low |
| Cozac (2016b) | Longitudinal | JBI Cohort | 8/11 | 8 | Low |
| Arnaldi (2017) | Longitudinal | JBI Cohort | 8/11 | 8 | Low |
| Wood (2021) | Observational | JBI Analytical | 7/8 | 7 | Moderate |
| Rea (2021) | Case-control | JBI Case-Control | 8/10 | 8 | Low |
| Mostile (2019) | Case-control | JBI Case-Control | 8/10 | 8 | Low |
| Zawiślak-Fornagiel (2023) | Case-control | JBI Case-Control | 8/10 | 8 | Low |
| Caviness (2015) | Longitudinal | JBI Cohort | 8/11 | 8 | Low |

**Table S7. Full search strings for all databases**

| Database | Search String |
| --- | --- |
| **PubMed** | ("Parkinson disease"[MeSH] OR "Parkinson's disease"[tiab] OR "Parkinson disease"[tiab] OR "idiopathic Parkinson"[tiab]) AND (electroencephalography[MeSH] OR EEG[tiab] OR "quantitative EEG"[tiab] OR qEEG[tiab] OR "spectral power"[tiab] OR coherence[tiab] OR connectivity[tiab] OR "dominant frequency"[tiab] OR "machine learning"[tiab] OR wavelet[tiab] OR TBPC[tiab]) AND ("1991/01/01"[PDAT] : "2025/03/31"[PDAT]) AND "humans"[MeSH Terms] AND English[la] |
| **Embase** | ('Parkinson disease'/exp OR 'Parkinson's disease':ti,ab OR 'Parkinson disease':ti,ab) AND (electroencephalography/exp OR 'eeg':ti,ab OR 'quantitative eeg':ti,ab OR 'qeeg':ti,ab OR 'spectral power':ti,ab OR 'coherence':ti,ab OR 'connectivity':ti,ab OR 'dominant frequency':ti,ab OR 'machine learning':ti,ab OR 'wavelet':ti,ab OR 'tbpc':ti,ab) AND [1991-2025]/py AND [humans]/lim AND [english]/lim |
| **Web of Science** | TS=("Parkinson disease" OR "Parkinson's disease" OR "idiopathic Parkinson") AND TS=(electroencephalography OR EEG OR "quantitative EEG" OR qEEG OR "spectral power" OR coherence OR connectivity OR "dominant frequency" OR "machine learning" OR wavelet OR TBPC) AND PY=(1991-2025) |
| **Scopus** | TITLE-ABS-KEY("Parkinson disease" OR "Parkinson's disease" OR "idiopathic Parkinson") AND TITLE-ABS-KEY(electroencephalography OR EEG OR "quantitative EEG" OR qEEG OR "spectral power" OR coherence OR connectivity OR "dominant frequency" OR "machine learning" OR wavelet OR TBPC) AND PUBYEAR > 1990 AND PUBYEAR < 2026 |
